# Case Report: Pseudoxanthoma elasticum

**DOI:** 10.12688/f1000research.21431.1

**Published:** 2020-01-09

**Authors:** Catarina Lucas, João Aranha, Isabel da Rocha, Domingos Sousa

**Affiliations:** 1 Family Health Unit, Baesuris Family Health Unit, Castro Marim, Faro, 8950-219, Portugal; 2 Dermatology Department, Hospital Distrital de Santarém, Santarém, Santarém, 2005-177, Portugal; 3 Personalized Health Care Unit, Personalized Health Care Unit of Penacova, Coimbra, Coimbra, 3360-205, Portugal; 4 Internal Medicine Department, Centro Hospitalar e Universitário do Algarve, Faro, Faro, 8000-386, Portugal

**Keywords:** Pseudoxanthoma elasticum, ABCC6 gene, retina angioid streaks, hypertension

## Abstract

Pseudoxanthoma elasticum (PXE) is a rare inherited disorder, characterised by a progressive mineralization and fragmentation of elastic fibres of the skin, retina and cardiovascular system. At an initial stage, the skin usually exhibits distinctive lesions and subsequently extra-dermal manifestations. The diagnosis is based on clinical manifestations, histological analysis of the lesions and genetic analysis.

This is a case report of a 12-year-old child complaining of painless, mildly itchy yellow papules in the cervical region with 1 year of evolution.

PXE is currently an incurable disease and has a favourable prognosis when cardiovascular and retinal complications are prevented and monitored.

## Introduction

Pseudoxanthoma elasticum (PXE), also known as Grönblad-Strandberg syndrome, is a rare inherited disorder presenting an autosomal recessive inheritance with a mutation in the *ABCC6* gene (ATP-binding cassette transporter C6), mapped in the chromosome 16. It has an estimated prevalence of 1/25.000 to 1/100.000 inhabitants and is 10 times more prevalent in women ^1, 2^.

The disease is characterised by a progressive mineralization and fragmentation of elastic fibres of the skin, retina, gastrointestinal and cardiovascular systems.

PXE results in a variety of signs and symptoms that vary in their number, type, and severity from person to person. Certain effects of PXE can cause serious medical problems, while others have less impact. Effects may include: skin changes, changes in the retina of the eye that may result in significant loss of central vision, changes in the cardiovascular system that may involve calcification of arteries and decreased blood flow in the arms and legs, and/or changes in the gastrointestinal system that may lead to bleeding in the stomach or intestines. At present, there is no way to predict the exact progression of the disorder for an individual ^2^. Some people have no skin lesions; others have no vision loss. Many people do not experience gastrointestinal complications or cardiovascular difficulties. A few have no manifestations of PXE except for a positive skin biopsy or irregular streaks resembling a blood vessel (angioid) in the retina of the eye ^1, 2^.

The diagnosis is based on major and minor criteria defined in 1994 taking into account the clinical manifestations described above ( Table 1), positive histological on Von Kossa stain for reticular dermis, family history and genetic analysis of the *ABCC6* gene ^1– 3^.

**Table 1.** Diagnostic criteria for Pseudoxanthoma elasticum (PXE) defined at the consensus conference in 1994 ^3^.

Major criteria
• Characteristic skin signs – Yellow cobblestone lesions in flexural areas • Characteristic histological features of lesional skin – Elastic tissue and calcium or von Kossa stains • Characteristic ophthalmologic features – Angioid streaks, peau d’orange maculopathy – In adults > 20 years old
Minor criteria
• Characteristic histological features of non-lesional skin – Elastic tissue and calcium or von Kossa stains • Family history of PXE in first-degree relatives

PXE is currently an incurable disease but has a favourable prognosis with appropriate follow-up by multidisciplinary teams.

## Case description

A 12-year-old Caucasian girl presented for a dermatology appointment in November 2016 due to sporadically painless, slightly itchy yellow papules in the cervical region with 1 year of evolution. These lesions remained stable throughout this period with no medication or treatment. The child had no pain complaints, or inflammatory signs on the lesions or around them, and did not have any associated symptoms. Previously she was a healthy child, was not taking any daily medication and had no relevant personal or family medical history of dermatosis.

**Figure 1.**
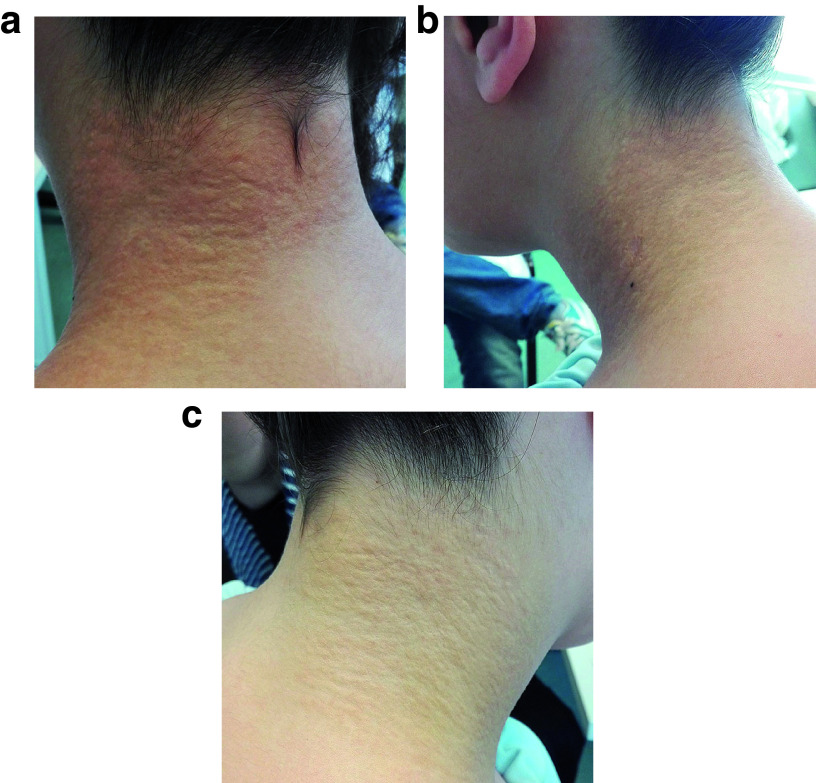
Photographs of the skin lesion taken from ( **a**) behind, ( **b**) the left and ( **c**) right side of the patient’s neck. Uneven, rough and yellow plaques can be seen in the cervical region.

On examination, painless, uneven, rough and yellow plaques without inflammatory signs in the posterior cervical region that merged bilaterally and symmetrically into the right and left side cervical region was found ( Figure 1). It was similar to a goose bump pattern, giving it a parchment-like skin appearance. The remaining integument had no changes.

No other abnormalities were found on the rest of physical examination.

In this appointment, the following exams were conducted: hemogram, general biochemistry with lipid profile and phosphorus-calcium balance and urinary sediment that were unremarkable. Moreover, a skin biopsy was promptly performed.

In the next appointment, still in November, the skin biopsy results revealed limited changes to the superficial/medium reticular dermis with a long strip of elastic fibre fragmentation. They were thick, granular, basophilic, with bizarre shapes between the collagen fibres with a normal appearance. There was no evidence of mineralization or deposits of mucin.

Following the investigation, further exams were conducted: electrocardiogram and echocardiogram, carotid and aortoiliac venous and arterial ultrasonography, retinogram with full ophthalmological examination (angioid streaks). The results were normal. There was the possibility of a genetic disorder, but the pathology specialist decided there was no need to perform a study of the *ABCC6* genes based on the clinical aspect of the lesions, which were highly characteristic of PXE.

The child has been having annual follow-up with ophthalmologic, paediatric cardiology and dermatology appointments. Until February 2019 no systemic manifestations were reported.

## Discussion

PXE is a rare inherited disorder, characterised by mineralization disturbances of the connective tissue with elastic fibre degeneration, mainly involving skin, eyeballs and cardiovascular system ^1– 4^.

At the consensus conference, in 1994, the PXE diagnosis criteria were defined ( Table 1) ^3^. The patient presented two of these major criteria: characteristic yellow cobblestone lesion in the skin and positive von Kossa stain.

Although the cutaneous manifestation is frequently flesh-colored to yellow macules or papules which progressively agglutinate into bigger plaques, and the affected skin that becomes lax and wrinkled, the clinical manifestation is variable and can occur at any age. At an early stage, the skin lesions are found on the right, left and rear sides of the neck and flexion creases, including armpits, inguinal region, popliteus region, and periumbilical area, as seen in this patient. The lesions may also affect the genital area and oral mucosa ^2, 4, 5^.

PXE ophthalmological manifestations include mainly angioid streaks due to Bruch membrane lesions (characteristic but not pathognomonic), deformed macular degeneration, retina pigmentation, choroidal neovascularization, haemorrhage and scar formation on the retina. For the ocular complications, vascular endothelial growth factor antagonists have been used to prevent neovascularization, thus reducing the occurrence of the most severe complication: loss of vision ^4, 6^.

Cardiovascular manifestations are a major cause of morbidity in these patients: hypertension, angina pectoris and intermittent claudication are some of the examples. Patients with PXE may also develop early atherosclerosis due to the mineralization of the internal elastic lamina of the blood vessels and lipid alteration with high density lipoprotein (HDL) cholesterol levels reduction in blood plasma and hypertriglyceridemia. This contributes to a higher incidence of acute myocardial infarction and cerebrovascular accident ^6^.

Histopathology exams in PXE patients reveal agglutination and mineralization of elastic fibres and fragmentation of the elastic fibres in the medium/deep dermis ^2, 7^.

PXE and papillary dermal elastosis share similar lesions. Therefore, a differential diagnosis shall be performed. At a histopathology level, papillary dermal elastosis with the Von Kossa stain is negative to calcium in elastic fibres. Moreover, there is no systemic involvement in papillary dermal elastosis ^7^.

The patient’s general practitioner shall be responsible for early detection of the ocular and cardiovascular complications of the PXE because the mineralization in the centre of the fibres predisposes the development of secondary hypertension. Therefore, the morbidity and mortality caused by the disease can be monitored and reduced.

To conclude, PXE is a rare disease, the recognition of which is important since it can cause systemic severe cardiovascular and retinal manifestations. The diagnosis is based on histology result. Patients can be further tested for a mutation in the *ABCC6* gene. The earlier the diagnosis, the sooner preventive measures and close observation can be adopted to prevent and control the possible adverse events caused by this disease.

## Consent

Written informed consent for publication of their clinical details and clinical images was obtained from the parent of the patient.

## Data availability 

### Underlying data

All data underlying the results are available as part of the article and no additional source data are required.
