# Supplementary material for: Case Report: Pseudoxanthoma elasticum
Source: F1000Res. 2020 Jan 9;9:9. [Version 1] doi: 10.12688/f1000research.21431.1 (PMC7372526; doi:10.12688/f1000research.21431.1)
Supplement: Peer review history

# Peer review: Reviewer response for version 1

**DOI:** 10.5256/f1000research.23605.r66220

**Published:** 2020-07-31

**Authors:** Wilko Spiering

**Affiliations:** 1 Department of Vascular Medicine, University Medical Center Utrecht, Utrecht University, Utrecht, The Netherlands

This case report reports on a 12 year old child with PXE. 

 I have the following comments:

- Some references used are outdated and even contains a non-English paper. Please update and replace.
- The male-female ratio is not correct. PXE is 2-3 times more prevalent in women.
- The diagnostic criteria used are from 1994. I would recommend using the updated criteria from Plomp *et al. *( http://www.ncbi.nlm.nih.gov/pubmed/20358627), published in 2010. ^1^
- The authors state that PXE ‘has a favourable prognosis with appropriate follow-up by multidisciplinary teams’. What is meant by favourable? Although recommended, it is not proven that multidisciplinary teams improve any outcome in PXE.
- I disagree with the decision of the pathology specialist that there was no need to perform a genetic analysis for mutations in ABCC6, or other genes like ENPP1 and GCCX. A genetic analysis always should be done. This obviously cannot be changed in the manuscript, but maybe the authors can make a statement on this.
- Eyeballs are not affected in PXE. Please change into Bruch’s membrane of the eye or retina.
- I would prefer to use vascular manifestations instead of cardiovascular as the heart is usually not more affected compared with healthy controls. Also, hypertension is probably not more prevalent in PXE.
- It is stated that there is a ‘higher incidence of acute myocardial infarction and cerebrovascular accident in PXE’. The reference (Hosen *et al.*) used for this statement is a histopathology paper and does not even discuss this. Cerebrovascular accidents, but not myocardial infarctions, are indeed increased in PXE. Please use an appropriate reference.
- It is stated that ‘Patients with PXE may also develop early atherosclerosis due to the mineralization of the internal elastic lamina of the blood vessels and lipid alteration with high density lipoprotein (HDL) cholesterol levels reduction in blood plasma and hypertriglyceridemia.’ Atherosclerosis is not more common in PXE, as this is an intimal disease with accumulation of cholesterol in the intima. The appropriate vascular manifestation in PXE is arteriosclerosis, with calcification of the internal elastic layer and media of the vessel wall. The lipid alterations described are pathophysiological not correct and should be deleted.
- Recent developments on potential treatments in PXE are lacking, e.g. etidronate (Kranenburg *et al.* 2018) and oral pyrophosphate (Väärämäki *et al.* 2019). ^1^^,^^2^ I would recommend adding this to the discussion.

Are enough details provided of any physical examination and diagnostic tests, treatment given and outcomes?

Yes

Is the case presented with sufficient detail to be useful for other practitioners?

Partly

Is sufficient discussion included of the importance of the findings and their relevance to future understanding of disease processes, diagnosis or treatment?

Partly

Is the background of the case’s history and progression described in sufficient detail?

Yes

Reviewer Expertise:

Vascular medicine, pseudoxanthoma elasticum, hypertension.

I confirm that I have read this submission and believe that I have an appropriate level of expertise to confirm that it is of an acceptable scientific standard, however I have significant reservations, as outlined above.

## References

[1] : Proposal for updating the pseudoxanthoma elasticum classification system and a review of the clinical findings. *Am J Med Genet A*.2010;152A(4) : 10.1002/ajmg.a.33329 1049-58 10.1002/ajmg.a.33329 20358627

[2] : Etidronate for Prevention of Ectopic Mineralization in Patients With Pseudoxanthoma Elasticum. *Journal of the American College of Cardiology*.2018;71(10) : 10.1016/j.jacc.2017.12.062 1117-1126 10.1016/j.jacc.2017.12.062 29519353

[3] : Pyrophosphate Treatment in Pseudoxanthoma Elasticum (PXE)-Preventing ReOcclusion After Surgery for Critical Limb Ischaemia. *Surgical Case Reports*.2019; 10.31487/j.SCR.2019.04.02 1-3 10.31487/j.SCR.2019.04.02 30673900

# Peer review: Reviewer response for version 1

**DOI:** 10.5256/f1000research.23605.r66222

**Published:** 2020-07-20

**Authors:** Annamaria Offidani

**Affiliations:** 1 Dermatological Clinic, Department of Clinical and Molecular Sciences, Polytechnic University of the Marche Region, Ancona, Italy

- The title is written in SEO terms.
- The text is clear and well written, the case is described in detail. Our response is to approve the manuscript as it doesn't present flaws or incomplete descriptions of the steps from diagnosis to management.
- It would be nice if the authors could add some details of dermoscopic characteristic features they saw as dermoscopy could substitute histopathology for early and confirmed diagnosis. ^1^

Are enough details provided of any physical examination and diagnostic tests, treatment given and outcomes?

Yes

Is the case presented with sufficient detail to be useful for other practitioners?

Yes

Is sufficient discussion included of the importance of the findings and their relevance to future understanding of disease processes, diagnosis or treatment?

Yes

Is the background of the case’s history and progression described in sufficient detail?

Yes

Reviewer Expertise:

Immunological skin diseases, psoriasis, atopic dermatitis, genetic and rare disease of the skin

I confirm that I have read this submission and believe that I have an appropriate level of expertise to confirm that it is of an acceptable scientific standard.

## References

[1] : Dermoscopy of pseudoxanthoma elasticum. *BMJ Case Reports*.2017; 10.1136/bcr-2017-221365 bcr-221365 10.1136/bcr-2017-221365 PMC569536329127123

# Peer review: Reviewer response for version 1

**DOI:** 10.5256/f1000research.23605.r66211

**Published:** 2020-07-17

**Authors:** Márta Medvecz

**Affiliations:** 1 Department of Dermatology, Venereology and Dermatooncology, Semmelweis University, Budapest, Hungary

This manuscript for a case report presents the well-documented case of a 12-year-old girl with PXE. In addition, the discussion section of the paper also includes a short review on PXE, highlighting systemic manifestations, histopathologic alterations, and management. However, there are some minor issues to be clarified by the authors:

1. A more informative title could be useful, e.g. one that implies that an interesting aspect of this case is that early skin lesions in a young patient are documented. 
2. It would raise the quality of the manuscript if, in addition to the description of the skin biopsy findings, histopathologic images could also be included. Also, for the histology, it should be mentioned, which stainings were performed. 
3. The necessity of genetic testing should be decided by a clinical geneticist and not a pathologist, as implied in the Case description of the manuscript. 
4. In the Discussion section, it can be debated if the patients’ general practitioner should be responsible for the early detection of systemic manifestation. PXE should be managed by a multidisciplinary team at specialized rare disease centers with sufficient expertise and experience. 
5. Certain papers cited by the manuscript are outdated or are not available in English. These should be replaced by citing more recent, high-quality publications.

Are enough details provided of any physical examination and diagnostic tests, treatment given and outcomes?

Yes

Is the case presented with sufficient detail to be useful for other practitioners?

Partly

Is sufficient discussion included of the importance of the findings and their relevance to future understanding of disease processes, diagnosis or treatment?

Yes

Is the background of the case’s history and progression described in sufficient detail?

Partly

Reviewer Expertise:

Genodermatoses

I confirm that I have read this submission and believe that I have an appropriate level of expertise to confirm that it is of an acceptable scientific standard, however I have significant reservations, as outlined above.

# Peer review: Reviewer response for version 1

**DOI:** 10.5256/f1000research.23605.r58434

**Published:** 2020-02-17

**Authors:** Olivier M. Vanakker

**Affiliations:** 1 Center for Medical Genetics, Ghent University Hospital, Ghent, Belgium

This case report describes a classic presentation of PXE in a young girl. 

 I have a few comments on the current manuscript:

1. The manuscript refers in the introduction and discussion to the detection of a mutation in ABCC6, but PXE is an autosomal recessive disease, so this should be mutations in ABCC6. 
2. PXE can also be caused by mutations in the ENPP1 gene. This should be added. 
3.  The difference between women and man is approximately 3:1, not 10:1
4. In the description of the ocular lesions, there should be a distinction between the angioid streaks due to Bruch's membrane abnormalities - which are almost always present - and retinal changes, i.e. a retinal dystrophy which can occur but is much more rare.
5. The idea of not analyzing ABCC6 when the histology is typical is not correct for two reasons:
  a. in its initial presentation - both clinical and histological - PXE can high resemble the PXE-like syndrome with coagulation factor deficiency due to GGCX mutations. The natural history and follow-up of these patients is however completely different. 
  b. identification of the causal mutations - either in ABCC6 or ENPP1 - will allow to detect subclinical patients in the family (as said, some have no skin lesions and angioid streaks do not have to become symptomatic immediately) as well as heterozygous carriers. The latter also have a higher cardiovascular risk and a higher risk for stroke and are candidates for primary prevention. 
  So every patient should have molecular analysis performed!
6. In the first paragraph of the discussion, the authors mention the eyeballs. But there is nothing wrong with the eyeballs themselves. 
7. PXE patients have media calcification in their arteries, which leads to arteriosclerosis, not atherosclerosis (which reflects intima calcification). 
8. The differential diagnosis is absolutely not complete: one should exclude the hemoglobinopathy-associated phenocopy, the PXE-like syndrome with coagulation factor deficiency, the PXE-like phenotype with pigmented retinopathy, ...

Are enough details provided of any physical examination and diagnostic tests, treatment given and outcomes?

Yes

Is the case presented with sufficient detail to be useful for other practitioners?

Partly

Is sufficient discussion included of the importance of the findings and their relevance to future understanding of disease processes, diagnosis or treatment?

Yes

Is the background of the case’s history and progression described in sufficient detail?

Partly

Reviewer Expertise:

Ectopic mineralization diseases

I confirm that I have read this submission and believe that I have an appropriate level of expertise to confirm that it is of an acceptable scientific standard, however I have significant reservations, as outlined above.
